# Poincaré Maps and Aperiodic Oscillations in Leukemic Cell Proliferation Reveal Chaotic Dynamics

**DOI:** 10.3390/cells10123584

**Published:** 2021-12-19

**Authors:** Konstantinos Adamopoulos, Dimitis Koutsouris, Apostolos Zaravinos, George I. Lambrou

**Affiliations:** 1 Biomedical Engineering Laboratory, School of Electrical and Computer Engineering, National Technical University of Athens, Heroon Polytechneiou 9, 15780 Athens, Greece; kadamopoulos@biomed.ntua.gr (K.A.); dkoutsou@biomed.ntua.gr (D.K.); 2 Department of Life Sciences, School of Sciences, European University Cyprus, Diogenis Str. 6, Nicosia 2404, Cyprus; a.zaravinos@euc.ac.cy; 3 Basic and Translational Cancer Research Center (BTCRC), Cancer Genetics, Genomics and Systems Biology Group, European University Cyprus, Nicosia 1516, Cyprus; 4 Choremeio Research Laboratory, First Department of Pediatrics, National and Kapodistrian University of Athens, Thivon & Levadeias 8, Goudi, 11527 Athens, Greece

**Keywords:** CCRF-CEM, Poincaré, Poincaré maps, chaos, chaotic mechanics, acute lymphoblastic leukemia, chaotic orbits

## Abstract

Biological systems are dynamic systems featuring two very common characteristics; Initial conditions and progression over time. Conceptualizing this on tumour models it can lead to important conclusions about disease progression, as well as the disease’s “starting point”. In the present study we tried to answer two questions: (a) which are the evolving properties of proliferating tumour cells that started from different initial conditions and (b) we have attempted to prove that cell proliferation follows chaotic orbits and it can be described by the use of Poincaré maps. As a model we have used the acute lymphoblastic leukemia cell line CCRF-CEM. Measurements of cell population were taken at certain time points every 24 h or 48 h. In addition to the population measurements flow cytometry studies have been conducted in order to examine the apoptotic and necrotic rate of the system and also the DNA content of the cells as they progress through. The cells exhibited a proliferation rate of nonlinear nature with aperiodic oscillatory behavior. In addition to that, the (positive) Lyapunov indices and the Poincaré representations in phase-space that we performed confirmed the presence of chaotic orbits. Several studies have dealt with the complex dynamic behaviour of animal populations, but few with cellular systems. This type of approach could prove useful towards the understanding of leukemia dynamics, with particular interest in the understanding of leukemia onset and progression.

## 1. Introduction

The answer to the argumentations between random and deterministic, was given shortly before the end of the 19th century by *Henri Poincaré*. It simply took several decades for its results to be properly interpreted. *Poincaré* proved that even in the simplest problems of Engineering and Astronomy, there are solutions (or trajectories) so sensitive to their initial conditions that makes their evolution over time completely unpredictable. Even the simplest deterministic systems, moving in a space of at least three dimensions, have areas where their solutions are so unstable that even minimal shifts in their initial state lead to huge changes in their evolution. *Henri Poincaré* worked on the three-body problem in 1889, during a contest on celestial mechanics. The three-body problem is related to the question on the stability of the solar system, in particular the n-body problem. In this contest, the winner was *Henri Poincaré* [1].

### 1.1. Acute Lymphoblastic Leukemia of Childhood

#### 1.1.1. Phenotypic and Molecular Characteristics of Leukemia

Childhood leukemia is the most common type of childhood cancer and represents clonal proliferation of transformed hemopoietic cells as a result of genomic and proteomic changes [2]. Further on, leukemia is a heterogeneous malignancy able to occur at different stages of lymphoblast maturation/differentiation.

There are two main categories of childhood leukemia: *Acute Leukemia* and *Chronic Leukemia*. Acute Leukemia can be subdivided further on, to *acute myeloblastic (AML)* and *acute lymphoblastic leukemia. Acute Lymphoblastic Leukemia (ALL)* is the most common pediatric malignancy. Leukemia stratification is placed on certain morphologic, immunophenotypic and cytogenetic criteria. Lymphoblasts share a common characteristic: both (B-cell ALL and T-cell ALL) rearrange their immunoglobulin heavy chain and *T-cell Receptor* genes respectively. Especially, in almost all cases of early pre-B-ALL (*common* ALL) the Ig heavy chain gene is rearranged. The rearrangement also takes place in other types but in a lower extent of about 40%. This property of leukemic cells is exploited in order to detect *Minimal Residual Disease* (MRD) [3] using the *Polymerase Chain Reaction method* (PCR). In addition, there are efforts for using Ig-heavy chain rearrangement as a prognostic and treatment directed factor [2]. One of the most important criteria in leukemia classification are chromosomal aberrations. Some of the most significant aberrations, known up-to-date, are *hyperdiploidy (n* > *50)*, the *TEL/AML1* t(12;21)(p13:22), the *BCR/ABL* t(9;22)(q34;q11.2), the *MLL* t(4;11)(q21;23), t(9;11)(p22;q23), t(11;19)(q23;p13) and the *E2A-PBX1* t(1;19)(q23;p13) [2,4], which are also considered to be strong prognostic factors. Treatment of childhood leukemia has accomplished tremendous progress. Up-to-date approximately 80–90% of all cases are cured and manifest complete remission, whereas approximately 10% do not respond to therapy and relapse [2,3].

Although 85–90% of all leukemic patients, reach complete remission, there is a percentage that relapses. Thus, the importance of accurate diagnosis, including the type or subtype of the disease, as well as the definition of prognosis are crucial to leukemia progression.

Among the two types of leukemia (B-ALL and T-ALL) T-cell ALL manifests a difficult medical problem. Five different molecular pathways have been previously identified, leading to T-ALL, involving activation of different T-ALL oncogenes; HOX11, HOX11L2, TAL1, LMO1/2, LYL1, LMO2, and MLL-ENL. Gene expression studies identified the activation of a subset of these genes (HOX11, TAL1, LYL1, LMO1, and LMO2) in a larger fraction of T-ALL cases than those harboring chromosomal translocations. In many cases, the abnormal expression of one or more of these oncogenes is biallelic, indicating upstream regulatory mechanisms. Overexpression of the HOX11 orphan homeobox gene occurs in approximately 5% to 10% of childhood and 30% of adult T-ALL cases. Patients with HOX11-positive lymphoblasts have an excellent prognosis when treated with modern combination chemotherapy, while high risk groups of early failure are included largely in the TAL1- and LYL1-positive groups. Expression profiling of genes of T-ALL lymphoblasts is especially needed for patients on modern combination chemotherapy trials to clearly distinguish the 10% to 15% of patients who fail induction or relapse in the first year of treatment. These high-risk patients would be ideal candidates for more intensive therapies in first remission, such as myeloablative regimens with stem cell rescue. Based on the rapid pace of research in T-ALL, made possible in large part through microarray technology, deep analysis of molecular pathways should lead to new and much more specific targeted therapies [5].

#### 1.1.2. Leukemogenesis

Leukemogenesis is recognized as a multi-step process characterized by genetic instability and alterations in gene expression. Identification of these molecular changes can provide numerous opportunities for discovery of biomarkers to be used for early detection of cancers and for identifying novel molecular targets for preventive or therapeutic drugs.

Chromosomal abnormalities detected at diagnosis are associated with a high percentage of childhood leukemias. The early detection of such aberrations is of significant importance, not only for the classification of the leukemic subtype, but also for the prognosis and therapeutic outcome of the patients. On the other hand, leukemias involving the *BCR-ABL* fusion gene, present in about 4% of children with ALL, are characterised by a high incidence of relapses, whereas leukemias involving the *TEL-AML1* fusion are associated with a good prognosis [6,7].

Studies on monozygotic twins with concordant leukemia [6,7,8,9] and retrospective scrutiny of neonatal blood spots (Guthrie cards) of leukemia patients [10,11,12] have demonstrated that for a significant number of childhood patients leukemia starts at the embryonic stage. Further scrutiny of Guthrie spots showed that over 70% of children with ALL up to 13 years of age had clonotypic markers of the disease at birth [10,12,13,14]; the molecular markers present at birth were exactly the same as the ones detected in the leukemic cells a few years later. In another study it was shown that the particular fusion (*TEL-AML1*) is also detected in healthy children at birth with a frequency of 1% [15]. This 1% represents 100 times the risk of ALL with *TEL-AML1* fusion gene, indicating that the frequency of conversion of the pre-leukemic clone to overt disease is low [16,17]. This conclusion, in connection with the fact that other subtypes of leukemia (e.g., the ones characterised by the *BCR-ABL* fusion or *MLL* translocations) do not show clonotypic markers at birth [18,19,20,21,22], has led scientists to believe that a gene fusion alone cannot be the causative effect for the development of leukemia. Therefore, post-natal exposure and additional genetic events are required for clinically overt leukemia to take place.

#### 1.1.3. Non-Linear Dynamics in Leukemia Proliferation

Acute Lymphoblastic Leukemia (ALL) is the most common malignancy of childhood. ALL has been extensively studied in all aspects of its biology, including, but not limited to, classification, patient stratification, drug resistance and cell proliferation. However, in the context of cell proliferation, to our knowledge, there are no previous studies investigating its dynamics. A very important tool for the investigation of such problems are immortalized cell lines. Some previous works have reported the use of cells lines in order to study non-linear dynamics. Previous works by Wolfrom et al., (1994, 2000, 2004) [23,24,25], Guerroui et al., (2005) [26] and Laurent et al., (2010) [27] has investigated the chaotic dynamics of hepatocellular carcinoma cells as well as bone marrow progenitor cells [23]. In addition, few works have highlighted the oscillatory nature of leukemic cell proliferation [28]. Finally, in a previous work we have also reported that we have found evidence of deterministic chaos in leukemia cells in vitro [29]. In vitro systems give the possibility of performing long-term experiments, isolating the system under study, thus reducing noise, both of which are not possible in in vivo systems.

### 1.2. The Dynamics of Cell Proliferation in Leukemia. An In Vitro Model

What are the dynamics of tumor cells before diagnosis? How does tumor progress from its ontogenesis to formation of a complete system? These questions cannot be answered due to a practical problem; it is almost impossible to study cell populations in living organisms, in a temporal manner. Especially, in the case of cancer this is impossible since both practical as well as bioethical restrictions prohibit us from understanding the cell proliferation dynamics of a neoplasm.

It is known that biological systems do not follow “conventional” ways of growth. Their behavior is not regulated by simple models but complex (although sometimes very simple functions can manifest very complex behavior). However, why is it so important to know the way biological cells behave as a system? In order to answer this question we have to draw a line between two classes of different in vivo systems. The first, consist of normal (physiological) biological systems i.e., cells that progress through time working as a system in order to accomplish a certain purpose (e.g., a physiological process, a homeostatic process). The second is pathological, in our case a tumorigenic system, which also progresses through time also as one sound system in order to accomplish another purpose (or maybe not, since it is possible that tumor growth could be completely stochastic). In the case of a tumor the only purpose that we can speculate is its survival. In both systems their temporal progression is complex. This means that the state of the system cannot be predicted by the preceding conditions.

A major problem, in examining biological samples, especially biopsies or rare samples, is that the investigation gives a *stationary* “*picture*” (spatial, steady-state) of the system under investigation. This, compared to dynamic observations as for example in time-dependent studies, does not provide information for the evolution of the system rather it gives a “*snapshot*” of the moment. For this reason, it is difficult to obtain useful information on the examined samples. This is mainly to the fact that the term steady-state does not apply to biological systems. Biological systems are in constant “turmoil” changing energy and mass with their environment and at the same time they change their time-dependent behavior with respect to the environmental stimuli. Further on, an extension of this problem raises another question; what is the trajectory that tumor cells follow, i.e., proliferate, from the point of their first emergence to the point of clinical presentation? Towards that question there are not many methods that can facilitate its answer. Towards that end, we have attempted to create a model that would allow the investigation of tumor proliferation dynamics from the point of emergence to the point of clinical presentation.

### 1.3. The Mathematical Model and Analysis

#### 1.3.1. A General Description of the Growth Model

Let a phenomenon being described by a function of the form:

(1) y=f(x)

If time is added to the system, then Equation (1) becomes:

(2) $$\frac{dy}{dt}=f(x),x\in \mathbb{R}$$

If the system can be described linearly then its initial conditions will determine its further progression and it will be simple to calculate present states from previous ones, iteratively. Thus, given the present state of a pathogenic condition (tumor in this case) it would be required to know, with infinitesimal precision, the initial conditions. However, this is not the case in tumorigenic systems.

A well-studied model of cell progression described by the logistic equation presents the relation of cell population and competition for nutrition. The logistic equation is given by:

(3) f(x)=kx(1−x), x∈(0,1)

where *k* is the proliferation constant of cells. The graphical representation of this function is nonlinear. Taking the iteration of Equation (3) it becomes:

(4) $$f(x_{n+1})=kx_{n}(1-x_{n}),x\in \mathbb{R}\wedge n\in \mathbb{Z}$$

Two fixed points can be found for Equation (4), 0 and 1 [30]. This is true as long as the value of *k* is constant. For *k = 2* the function still has a linear form. However for *k* = 2.90, *k* = 3.22 and *k* = 3.93 the function manifests a totally different behavior [30]. In addition, when iterating Equation (4), for values of *k*: k∈[1,3,99] the system manifests a complex dendrogram [30]. Thus, it is obvious that a very simple function can manifest a very complex behavior. The bifurcartions manifested by the function for *k:* k∈[1,3,99], represent the change in cellular status [30]. Each bifurcation declares a decision for life or death, i.e., in cellular terms apoptosis or necrosis.

As *x* approaches 1 *y* approaches 0, which is expected since the competition for nutrients reaches its maxima and cells start to die instead of proliferating. In addition, the factor of volume (i.e., space for growth) is introduced in our study, which will be shown to play an important role in growth dynamics. In in vivo systems this is already known, as for example in the case of CNS tumors. Space is a factor which influences tumor growth and determines its behavior as far as its survival is concerned.

In the tumor setting, knowing the bifurcations of the system is of crucial importance since if the bifurcation diagram was known then it would be easier to “hit” the disease at those points where it turns itself to apoptotic pathways enhancing or even better amplifying the cause of apoptosis. The study of such systems in vivo is impossible since it cannot be estimated when exactly the first carcinogenic cells appeared in an organism. The reason for this in thermodynamic terms is that the information *I* for the system is described as $I(x,t),x\in \mathbb{R}\wedge \underset{t\to \infty }{\lim}I(x,t)\to 0$. In addition, as time progresses the entropy of the system increases as S(t)→∞. For that purpose, in vitro systems are the most suitable, for they can be maintained for long periods of time and their behavior can be studied.

#### 1.3.2. Dynamic Systems

A dynamic system is defined as any set of interacting physical, chemical, biological variables that evolve over time following specific laws [31,32]. Its only independent variable is time, which can be either continuous or discrete. For a continuous-time dynamic system to be studied, usually (ordinary) differential equations are required, while a discrete-time dynamic system is described by difference equations. The fact that the independent variable is discrete, means that we do not observe it constantly, but at regular intervals. Thus, let an *N*-dimensional space of dependent variables *x_k_*(*t*), for *k* = 1, 2, …, *N*, which have as their only independent variable time *t* and are components of the vector:

(5) $$x(t)=(x_{1}(t),x_{2}(t),\ldots ,x_{N}(t)),t\in I=(a,b)\;\mathrm{Dynamic}\;\mathrm{continuous}-\mathrm{time}\;\mathrm{system}$$

The time evolution of these vectors is given by a system of differential equations as follows:

(6) $${\dot{x}}_{k}=f_{k}(x,t),k=1,2,\ldots ,N$$

Such an example of a continuous-time dynamic system is the *Lotka-Volterra* equations. Let *x*_1_*(t)* and *x*_2_*(t)* are the populations of two different species, which grow, interact and compete. Their state can be described by a series of equation systems:

(7) $$\left\{\begin{array}{l} {\dot{x}}_{1}=ax_{1}-x_{1}x_{2}\\ {\dot{x}}_{2}=ax_{2}-x_{1}x_{2}\\ \begin{array}{c} .\\ .\\ . \end{array}\\ {\dot{x}}_{N}=ax_{N}-x_{1}x_{2} \end{array}\right\}\;\mathrm{where}\;a,\;b>\;0$$

The study of these equations can give an estimation of the population growth, as well as predict the system’s state. Similarly, for the discrete time system it is:

(8) $$x_{n}=x(t_{n})=(x_{1,n},x_{2,n},\ldots ,x_{N,n}),x_{k,n}=x_{k}(t_{n})$$

The temporal evolution of the system can be given by a system of equations such as:

(9) $$\begin{array}{l} x_{n+1}=g(x_{n})\\ x_{k,n+1}=g_{n}(x_{n}),k=1,2,\ldots N \end{array}$$

The independent variable *n* counts the time intervals, while the function *g* defines the laws of its evolution.

#### 1.3.3. Lyapunov Exponent

The *Lyapunov* exponent got its name from the Russian mathematician, *Aleksandr Mikhailovich Lyapunov* (1857–1918) and it is used for the investigation of a system’s dependence on its initial conditions. In the case of our cellular proliferating system, which follow the logistic equation, their progression over time could be described as:

(10) $$\Delta_{n}=\left|x_{n+1}-x_{n}\right|=\Delta_{0}e^{\lambda n}$$

where, Δ_0_ is the difference at time 0 (that would be Δ_1_* − *Δ_0_), *x_n_* is the population at time *t* and *x_n+_*_1_ is the population at time *t* + 1. The sensitivity of the system to its initial conditions can be quantified by the introduction of the *Lyapunov* exponent *λ*. The exponent calculates the sensitivity to initial conditions by actually comparing the trajectories of the same system for times *t* and *t* + 1. Assuming the *Napierian* logarithm:

(11) $$\ln\left(\frac{\Delta_{n}}{\Delta_{0}}\right)=n\lambda$$

For very small $\Delta_{0}$→0 it can be written:

(12) $$\left\{\begin{array}{l} \lambda =\frac{1}{n}\ln\left(\frac{dx\prime n}{dx}\right)\\ \lambda =\underset{n\to \infty }{\lim}\frac{1}{n}\sum_{i=0}^{n-1}\ln\left|x\prime \right| \end{array}\right\}$$

Thus, the exponent *λ* can estimate the degree of “*hyper-stretching*” for each iteration in a trajectory. If *λ* > 0, it suggests the expansion of the phase-space of a trajectory, meaning that its neighboring points separate rapidly, while a negative exponent (*λ* < 0) suggests “shrinking” (or attracting) of the phase-space of all points in a trajectory [33]. If a system is “attracted” to a fixed point, then all *Lyapunov* exponents are found to be negative, since the system evolves to the inside to a final fixed point or equilibrium. An attractor manifesting periodic behavior has exactly one exponent equal to zero and all others are negative. Yet, a strange attractor has at least one exponent positive and this implies a chaotic behavior [33]. These statements can be summarized as [33]:

(13) *λ*_i_ < 0, i = 1,2,…,n: Fixed point. System reaches equilibrium *λ*_1_ = 0 and *λ*_i_ < 0, i = 2,…,n: Periodic. System oscillates periodically *λ*_1_ = 0, *λ*_2_ = 0 and *λ*_i_ < 0, i = 3,…,n: Torus. At least one *λ*_i_ > 0, i = 1,2,…,n: System is chaotic.

Many methods have been proposed for the calculation of *Lyapunov* exponents and it is considered to be a tedious task [34,35]. In our case, given the function, which we have based our model on, we used for the approximate estimation of *Lyapunov* parameters the following definition: Let *f* be a smooth map on ℝ. The *Lyapunov* number *L*(*x*_1_) for the orbit {*x*_1_,*x*_2_,…,*x_n_*} is defined as:

(14) $$L(x_{1})=\underset{n\to \infty }{\lim}{\left(\left|f\prime (x_{1})\right|\ldots \left|f\prime (x_{n})\right|\right)}^{\frac{1}{n}}$$

If the limit exists, then the Lyapunov exponent *λ* is defined as:

(15) $$\lambda (x_{1})=\underset{n\to \infty }{\lim}\frac{1}{n}\left(\ln\left|f\prime (x_{1})\right|+\ldots +\ln\left|f\prime (x_{n})\right|\right)$$

#### 1.3.4. Poincaré Maps

*Poincaré* maps, where named after *Henri Poincaré*, to whom we referred in the introduction of this work. The basic property of these maps is that it gives a different perspective on continuous complex functions, whose trajectories, are very complex to investigate. Thus, instead of studying the whole trajectory/graph of a phenomenon, it is enough to find the points of intersection or passage of the graph from a two-dimensional plane. Let a graph *S_f_* of the function *f* that intersects the plane at two points *A* and *B*. If *A* intersects the plane for the *k*^th^ time, and *B* intersects the plane for the *k*^th+1^ time, then it can be proven that there is a graph such as:

(16) G:G(A)=B

**Figure 1:**
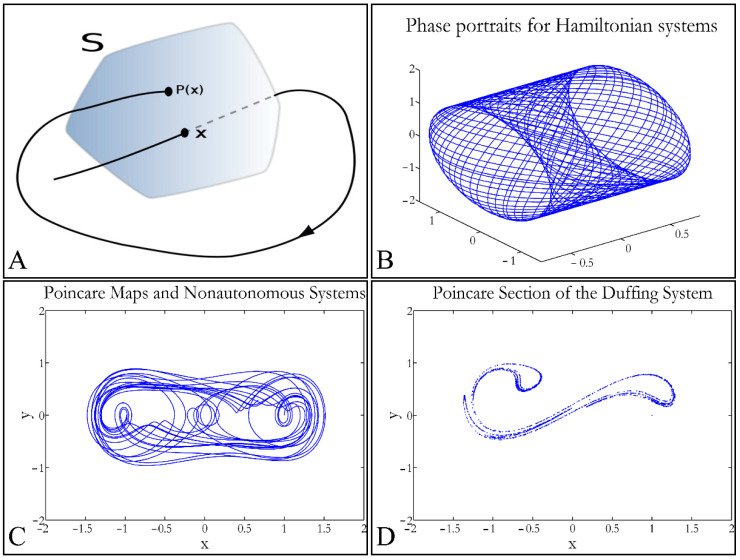
Examples of *Poincaré* maps. A trajectory intersects a plane at points *x* and *P*(*x*), where it is *x* = *P*(*x*) (**A**) (reproduced from https://en.wikipedia.org/wiki/Poincar%C3%A9_map, accessed on 24 May 2021). The phase portrait of a *Hamiltonian* function (**B**), with the respective *Poincaré* map (**C**), as well as the *Poincaré* map of a Duffing system (**D**) are presented.

In that way a dynamic system of *n* dimensions is transformed into a system of *n* − 1 dimensions. The following representation (Figure 1) manifests some interesting examples, as well as the *Poincaré* cross-section diagrammatically. *Poincaré*’s overall work on periodic solutions of differential equations has been extremely important and consists of the basis of many achievements to date. *Poincaré*’s fundamental idea was that instead of studying the whole trajectory (orbit), he would focus on its traces (sections) on a plane perpendicular to the orbit [33].

Thus, a *Poincaré* map is defined as the intersection of a periodic orbit in the phase-space of a continuous dynamic system, being transversal to the flow of the system. In particular, the map of the continuous system intersects the phase-space with a period of [33]:

(17) $$T=\frac{2\pi }{\omega }$$

The map transforms a continuous trajectory of points to points, which yet retain the momentum of the initial flow. The general equation for a map can be described as:

(18) $$\dot{x}=f(x),U\in {\mathbb{R}}^{N}\to {\mathbb{R}}^{N}$$

If a function has a flow *φ_t_*, with a period *T*, then it can be proven that *φ*(*t + T*, *x*_0_) = *φ*(*t*, *x*_0_). If a transversal cross-section (*Σ*) is taken to the function’s flow then a *Poincaré* map *P*(*x*), is described as *P*(*x*): *V* ∈ *Σ*→*Σ*, which correlates the vector $\bar{x}$, in space *V* with a point *P*($\bar{x}$) for each intersection [33]. Therefore, when the function’s flow crosses the transversal plane for the first time, it moves on and crosses the plane in a second point, then on a third, a fourth and so on. This process is mapped through the operator *P* such as:

(19) $$x_{t}=kx_{t}(1-x_{t})=0\Rightarrow x_{t}=0,x_{t}=\frac{R-1}{R}$$

where (*x*’,*y*’) is the recurrence intersection of point (*x*,*y*). For every successful recurrence, it can be proven that:

(20) $$\left(x_{2},y_{2})=P(P(x_{0},y_{0})=P^{2}(x_{0},y_{0}\right)$$

Thus, it is proven that $\left(x_{n},y_{n})=P^{n}(x_{0},y_{0}\right)$.

When the point where *P*(*x*) can mapped to itself, such as *P*(*x*_0_) = *x*_0_, this is called an equilibrium point [33]. This was the solution invented by *Poincaré*, to solve the three-body problem. *Poincaré* “invented” a plane, perpendicular to the trajectories of the bodies, which would describe the orbits on a two-dimensional plane. The first advantage of his conception was that it reduced the dimensions of the problem by 1. Thus, if three bodies (or planets) would follow *Newtonian* dynamics then their mapping would be manifested by single points on a plane (which is actually the phase-space of the trajectories). On the contrary, trajectories that do not follow predictable dynamics would be represented by the mapping of infinitesimally large number of points on a plane. Therefore, complex trajectories would also manifest complex *Poincaré* maps. From his observations, *Poincaré* found that there are trajectories that change their behavior due to small changes in their initial conditions, thus introducing chaos [33].

### 1.4. Aim and Objectives

In the present work we have attempted to provide further evidence based on an experimental model, that leukemic cell proliferation follows chaotic dynamics. Further on, we have attempted to prove that leukemic cell proliferation can be described by *Poincaré* maps. This study could provide insight on the dynamics, leukemia cells follow from the point of initiation (that would be the time, the first leukemic cells appear) to the point of clinical presentation. Towards that end, we have used in-house experimental data of in vitro cell proliferation of the T-cell leukemic cell line CCRF-CEM. In addition, we have used the approach of *Poincaré* recurrence maps in order to prove our concept.

## 2. Materials and Methods

### 2.1. The Cellular System

The system studied consisted of leukemic cells from an established cell line. The CCRF-CEM (T-Acute Lymphoblastic Leukemia (ALL)) cell line was used as the model, obtained from the European Collection of Cell Cultures (ECACC, Salisbury, United Kingdom). The CCRF-CEM cell line, a CD4+ [36] and CD34+ presenting cell line [37], was initially obtained from the peripheral blood of a 2 year old Caucasian female, with a karyotype of 46 XX. It was diagnosed as lymphosarcoma which progressed later on to acute lymphoblastic leukemia [38]. The child has undergone irradiation therapy and chemotherapy prior to obtaining the cell line. Although remission was achieved at various stages, the disease progressed rapidly [38]. The cell line has been observed to undergone minor changes after long-term culture, except for the presence of dense granules in the nucleoli [39], making it an ideal model system for long-term studies. Finally, the CCRF-CEM cell line has been reported to manifest autocrine catalase activity which participates to its mechanisms of growth and progression [40]. The CCRF-CEM cells give an excellent model of avascular growth. In addition, we assume that extracellular signal transduction takes place autocrinicaly.

Cells were grown with RPMI-1640 medium (Invitrogen, Carlsbad, CA, USA), 10% FBS (Invitrogen, Carlsbad, CA, USA) and 0.1 × Streptomycin/Penicillin (Invitrogen, Carlsbad, CA, USA) at 37 °C, 5% CO_2_ and ~100% humidity. Cells were cultured in 75 cm^2^ flasks in total medium volume of 25 mL. Cells were seeded at an initial concentration of 20 cells/μL and ~200 cells/μL and were fed at regular intervals thereafter. Medium changes took place by centrifugation at 1000 rpm for 10 min, the supernatant was discarded and the remaining cells were rediluted in 25 mL media and were allowed to grow.

### 2.2. Cell Population Measurements

**Figure 2:**
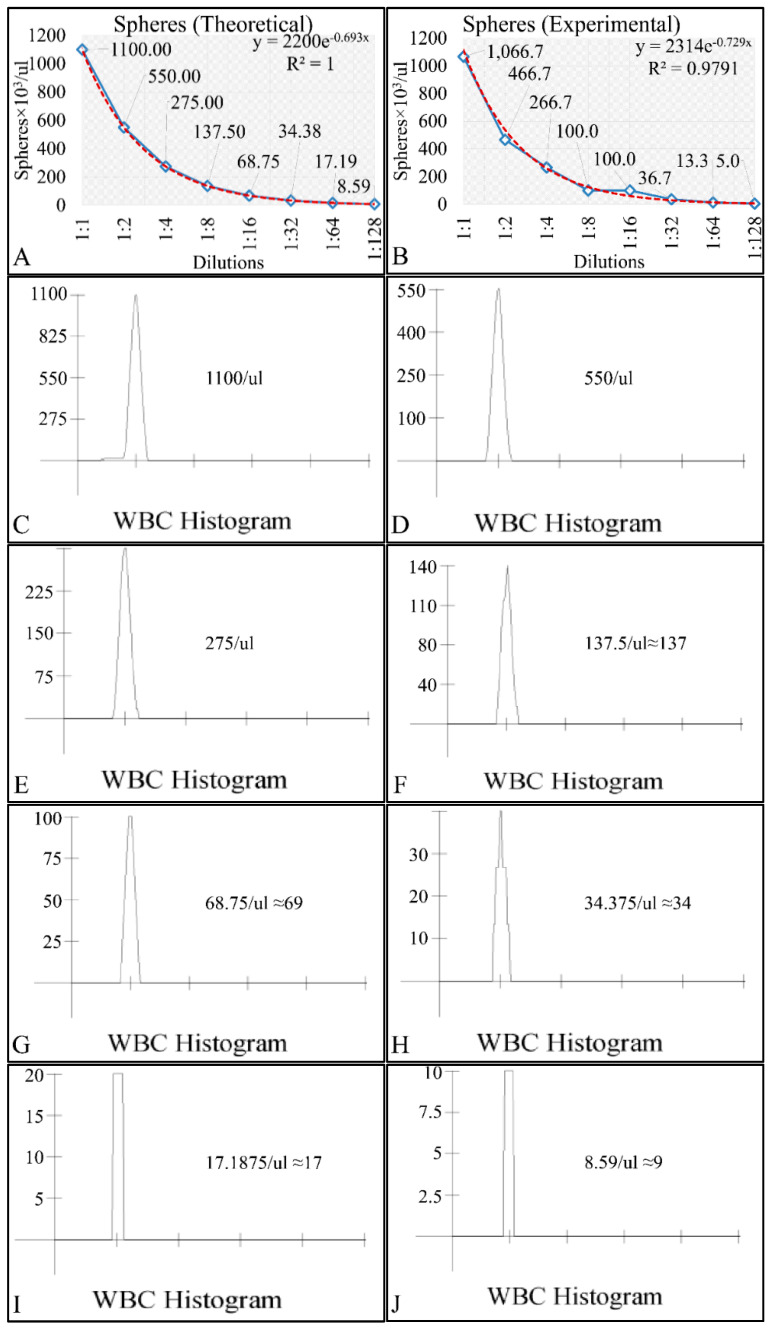
The calibration measurements with the Coulter counter, investigating the sensitivity of the instrument. In particular, the theoretical (**A**) and experimental (**B**) regressions of the dilution series are presented. Further on, the actual coulter-counter measurements are presented for sphere concentrations of 1100 spheres/μL (**C**), 550 spheres/μL (**D**), 275 spheres/μL (**E**), 137 spheres/μL (**F**), 69 spheres/μL (**G**), 34 spheres/μL (**H**), 17 spheres/μL (**I**) and 9 spheres/μL (**J**).

For the study of the growth dynamics of the cell culture system an experimental setup has been developed where cells seeded at the aforementioned concentration were measured at least every 48 h and every 3–5 days media was being renewed. For the measurements, 200 μL from each flask was taken and measured with a NIHON KOHDEN CellTaq-α hematology analyzer. The applied method is reported to manifest precision at the 1.21% coefficient of variance (CV) [41,42]. In addition, from in-house experiments we have performed dilution-series experiments, in order to calibrate the Coulter counter and confirm that the instrument was able to detect low populations (especially <100 cells/μL). Calibration was performed with spheres resembling leukocytes. Spheres of 10um in diameter were obtained from DAKO (DAKO Fluorospheres, DAKO Denmark Inc.), with initial concentration of 1.1 × 10^6^ spheres/μL. Initially, 1ml of the sphere solution was obtained and a 1:10 dilution was performed, in order to obtain a concentration of 1100 spheres/μL. Further on, a series-dilution was performed ranging from 1100 spheres/μL down to ~9 spheres/μL. The theoretical (Figure 2A) and experimental (Figure 2B) concentrations were measured and recorded. Since the Coulter counter was not able to represent a value <100 particles/μL, we have evaluated all dilutions using the histograms provided by the manufacturer. In particular, the histograms for all dilutions from 1100 spheres/μL to 9 spheres/μL are presented in Figure 2C–J. The reason for this type of evaluation, was to examine whether the instrument could detect much diluted sphere solutions and thus diluted cell populations.

**Figure 3:**
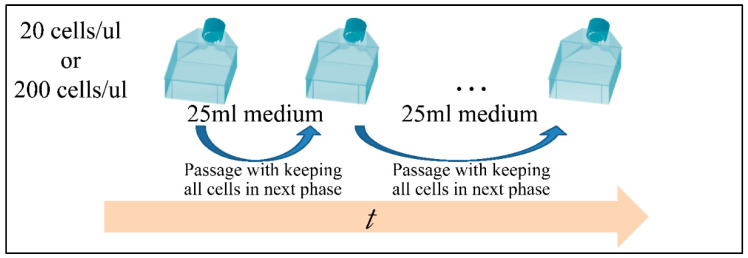
The experimental setup of the growth model [43].

All measurements were performed based on the experimental setup depicted in Figure 3. The experiments were performed in quadruplicates, yet with a particular setup. A set of experiments was performed for the total time period of approx. six months, and the duplicates were used as technical replicates. At the end of this experimental period, the experiment was repeated for another six months, which actually consisted of the biological replicate. All results presented, are the mean ± stdev of all replicates, both technical and biological.

### 2.3. Cell Viability Assays

In order to validate the viability of our model, we have performed random samplings throughout the experimental course, and samples were further analyzed with flow cytometry (FC500, Beckman Coulter Inc.). Cells were evaluated for their viable, apoptotic and necrotic content using the Propidium Iodide (PI)/AnnexinV method (Vybrant apoptosis kit, Molecular Probes Inc.). The AnnexinV was stained by Alexa fluorochrome (466 nm emission). Further on, cell cycle analysis was also performed using the Ethanol/RNAse/PI as previously described [44].

### 2.4. Microscopic Evaluation of the Growth Model

**Figure 4:**
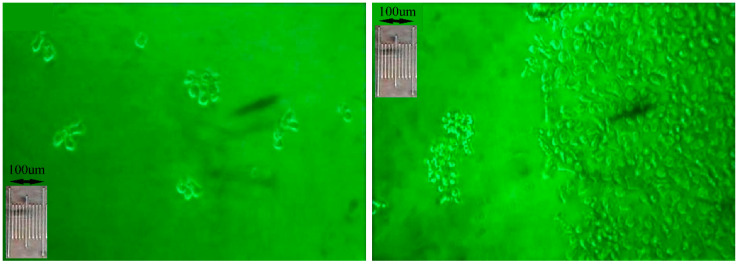
CCRF-CEM morphology during the experimental setup. Image acquisition was performed with an inverted microscope and a polarized light filter (magnification ×100). The micrometer presented is a Nikon micrometer, type MBM 11100 (stage micrometer type A), of 1mm total length and graduations of 0.01 mm (=100 μm). The micrometer is in the same magnification (×100) as the microscopy image [43].

During the course of the experiment, we have evaluated cellular condition with microscopy. Microscopic images were taken with Nikon TMS inverted microscope, optical condenser and lenses ×10, ×20 και ×40. In addition, a CCTV camera installed on the microscope (JVC Digital Camera TK-421C-EG), was used for image capture connected to a computer (Intel Celleron, RAM 256 MB). A representative microscopic image is presented in Figure 4 [43].

### 2.5. Data Analysis

Data have been initially collected with the Microsoft Excel^®^ for preprocessing. Data were treated as time-series and in particular, we have studied the cell populations with respect to the cell population per volume unit (μL), to the total cell population, as well as with respect to the extrapolation of the population to a single volume. Further on, the phase-space of the time-series has been used and the *Poincaré* mapping was estimated. In addition, we have calculated the *Lyapunov* exponents for the time-series trajectories.

## 3. Results

### 3.1. Time-Series

**Figure 5:**
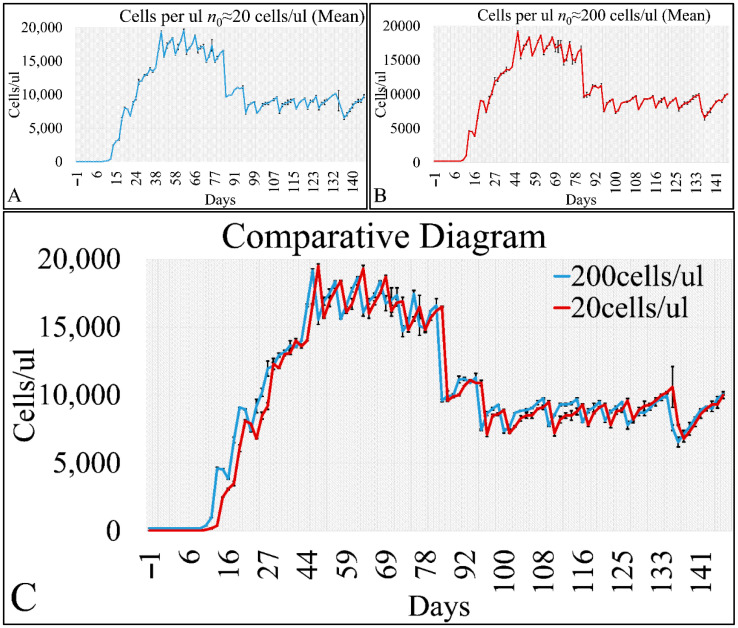
The time-series presentation of the cell population CCRF-CEM with respect to cell concentration (cells/μL) and in particular, for initial cell population of ~20 cells/μL (**A**), ~200 cells/μL (**B**). Further on, the two time-series are presented together in one diagram for comparison (**C**) (*n*_0_: the initial population).

The first step in describing the cell population data was to present them as time series. Cell population with respect to concentration per microliter (μL) appeared to be governed by oscillations. In particular, we have measured the cell population with initial conditions of *n*_0_ ≅ 20 cells/μL (Figure 5A) and *n*_0_ ≅ 200 cells/μL (Figure 5B). In addition, we have superimposed the two time-series and it appeared that the two curves progressed similarly, but with slight divergence (Figure 5C). The two experiments manifested small typical errors, indicating that there was a good repeatability in the experimental setups, which we also examined by regressing cell proliferation data with known growth models (Figure S1).

**Figure 6:**
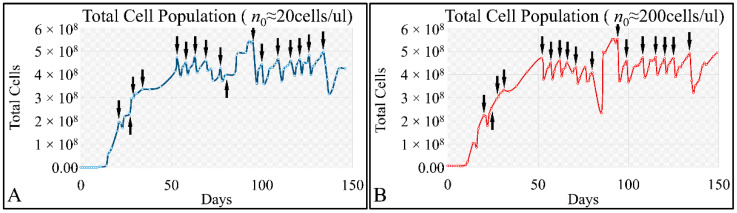
The time-series presentation of the cell population CCRF-CEM with respect to total cell concentration for initial cell populations of ~20 cells/μL (**A**) and ~200 cells/μL (**B**) (*n*_0_: the initial population. The arrows indicate the time-points of cell culture media replacement).

Similarly, the time-series of the total cell population manifested oscillatory dynamics for both initial cell populations i.e., 20 cells/μL (Figure 6A) and 200 cells/μL (Figure 6B).

### 3.2. Cell Viability

**Figure 7:**
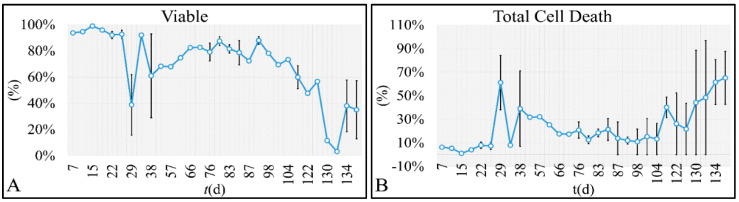
The time-series presentation of the cell population’s viability (**A**) and total cell death (**B**) of the CCRF-CEM cells.

In random time intervals we have evaluated the cell viability, in order to confirm that first of all viable cells were present, as well that cells were proliferating. Total cell death was described as the sum of both the apoptotic and necrotic cells. Interestingly, both viability (Figure 7A) and total cell death (Figure 7B) manifested oscillatory behaviors. Viable cells reached a minimum after 133 days, to rise again until the end of the experiment (Figure 7A). Similarly, total cell death manifested an increasing tendency from the beginning to the end of the experiment (Figure 7B).

### 3.3. Cell Cycle

**Figure 8:**
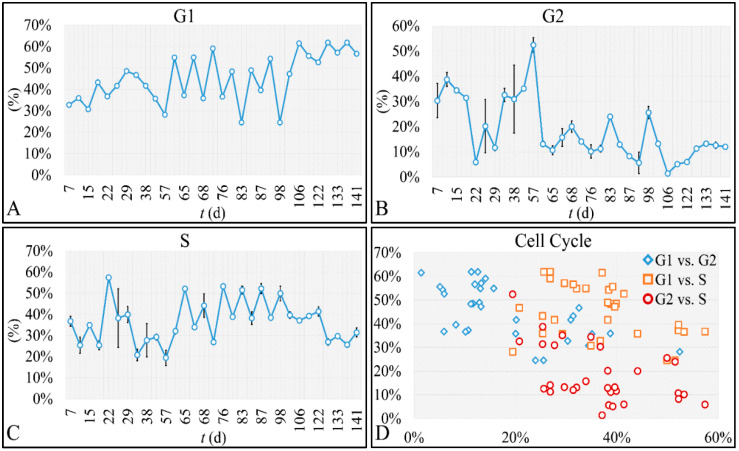
The cell population’s cell cycle. The cell cycle of our cellular system manifested oscillatory patterns for G_1_ (**A**), G_2_ (**B**) and S-phase (**C**). In addition, a scatter plot of the three cell cycle phases in all combinations showed a discrete separation of the three cell cycle phases (**D**).

Further steps into the analysis of our cell population included the investigation of the cell cycle, which would confirm that cells were actually proliferating. We have found that all cell cycle phases followed oscillatory dynamics and in particular the G1 phase (Figure 8A), the G2 phase (Figure 8B) and the S-phase (Figure 8C). In addition, we have performed a scatter plot of the combinations of all cell cycle phases and showed that all phases can be distinctively viewed (Figure 8D). Aside the cell proliferation we have investigated in the present work, cell cycle appears to follow its own dynamics, which will be the subject of a future investigation.

### 3.4. The Phase-Space of Cell Proliferation

**Figure 9:**
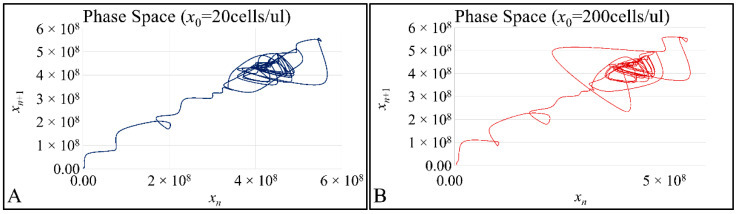
The phase-space for *x*_0_ = 20 cells/μL (**A**) and *x*_0_ = 200 cells/μL (**B**).

An interesting observation concerned the fact that despite the difference in the initial population, cell proliferation converged with small differences. We have examined the phase-space of the two experimental setups, and we have expected not to find orbital trajectories, but a “waltz” of traversing orbits. This was true for both initial populations i.e., *x*_0_ = 20 cells/μL (Figure 9A) and *x*_0_ = 200 cells/μL (Figure 9B). Thus, this was an indication that cell proliferation followed a chaotic orbit.

### 3.5. Lyapunov Exponents

**Figure 10:**
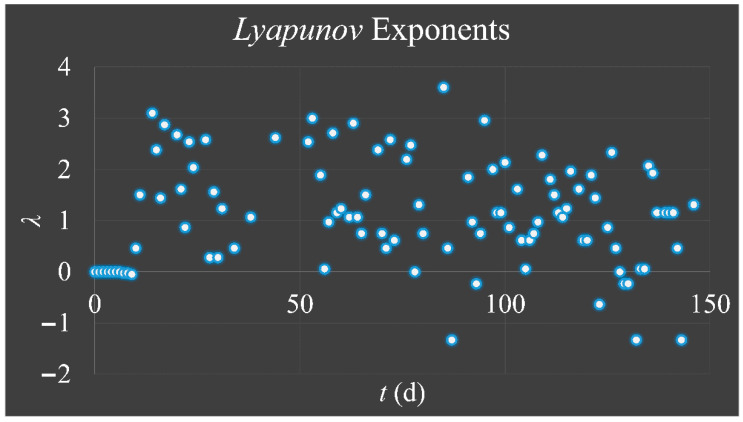
Calculations of the *Lyapunov* exponent (*λ*) for initial population 20 cells/μL and 200 cells/μL. We have calculated the Δ_0_ as Δ_0_ = |*x*_0_ − *x*_0′_|, where *x*_0_ = 20/max and *x*_0′_ = 200/max. The maximum population was considered to be the max population reached for each experiment in cells/μL.

In order to examine the presence of possible chaotic trajectories, we calculated the Lyapunov exponent. From our calculations it has been showed that the majority of the exponents was greater than zero (Figure 10), which was an indication of chaotic behavior since for a chaotic orbit one exponent greater than zero is sufficient to indicate chaos.

### 3.6. The Phase-Space of Cell Proliferation II

**Figure 11:**
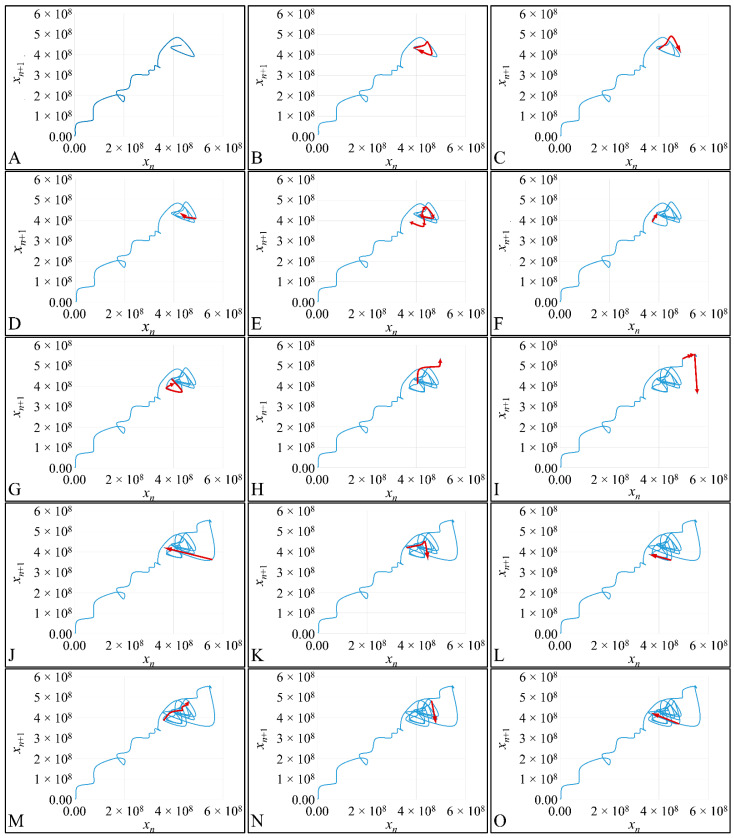

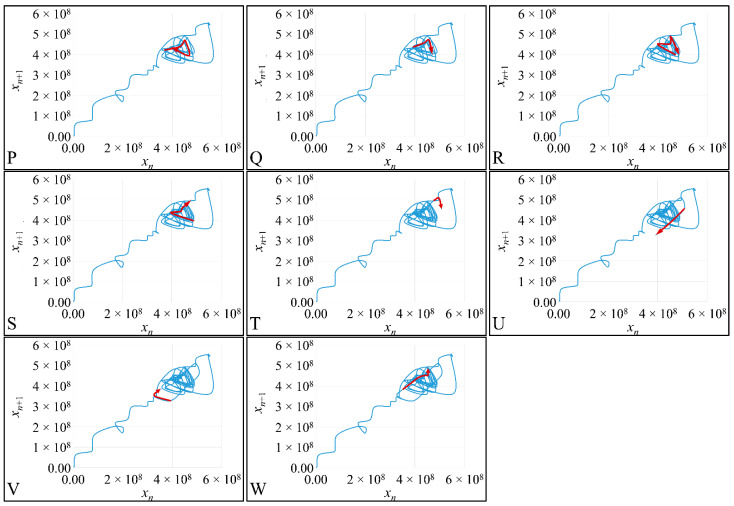
The phase-space steps of the experiment for *x*_0_ = 20 cells/μL. The red arrows indicate the course of the trajectory step-by-step. The *x*-axis corresponds to the population of cells at time t and the *y*-axis corresponds to the population of cells at time *t* + 1. Each of the subfigures (**A**–**W**) presents the phase-space recurrences from the first (**A**) to the 23rd (**W**).

The first step in the investigation of applying a Poincaré map to the proliferation phase-space we have analyzed the spatial trajectories for one of our experimental setups. In particular, we have followed, step-by-step, the trajectory of the proliferation for initial population 20 cells/μL (Figure 11) (we have also created a video, in which we showed the movement of the trajectory. Please refer to Supplementary File Video S1.mp4). This type of analysis allowed us to observe that the phase-space trajectory returns around points, which could probably be on the diagonal of the phase-space.

### 3.7. The Phase-Space of Cell Proliferation III

**Figure 12:**
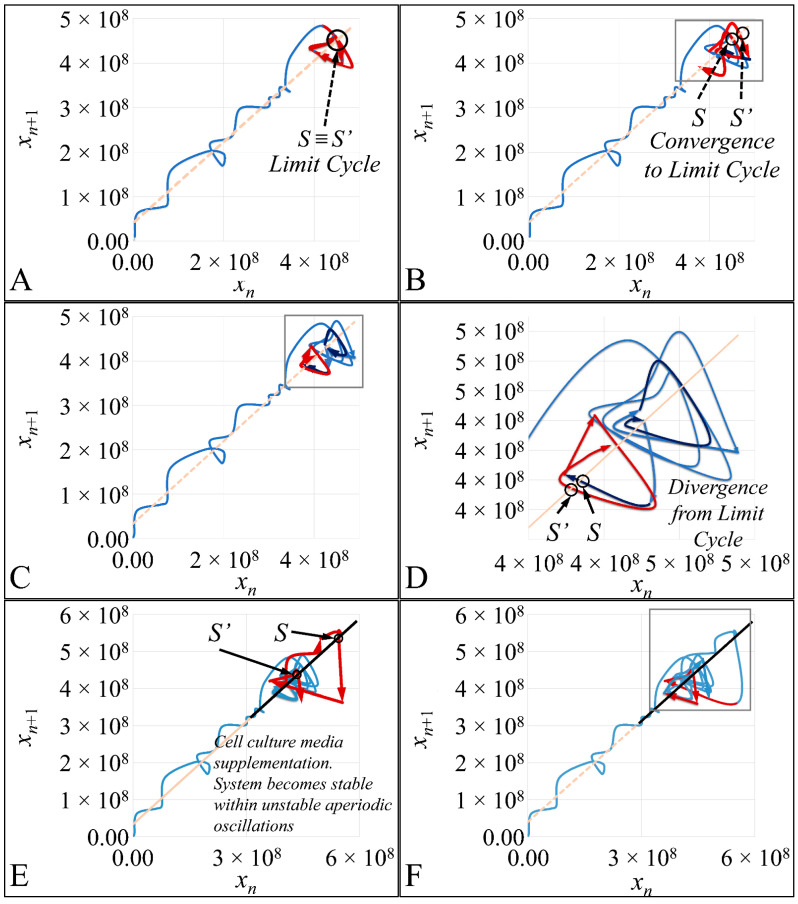

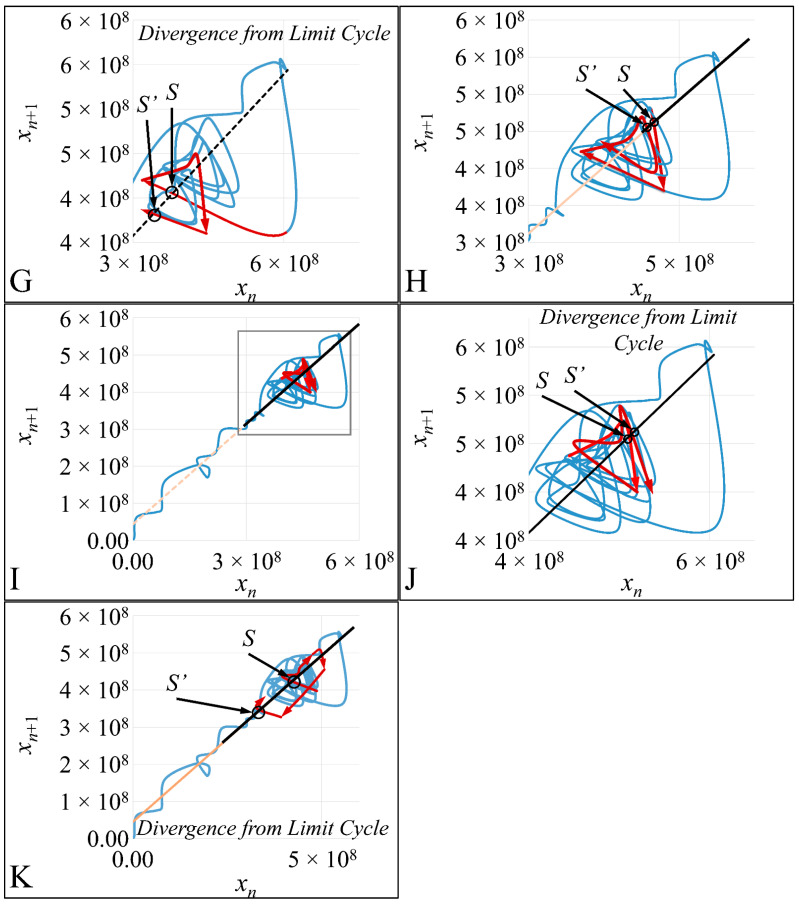
The phase-space of the experiment for *x*_0_ = 20 cells/μL. The red arrows indicate the course of the trajectory step-by-step. In addition, in all subfigures the diagonal is drawn, which shows the *Poincaré* map crossings. The *x*-axis corresponds to the population of cells at time *t* and the *y*-axis corresponds to the population of cells at time *t* + 1. The system converged in the first convolution of the trajectory (**A**,**B**) as well as after cell culture media supplementation (**E**). In the other cases (**C**,**D**,**F**–**K**) diverged from equilibrium.

Based on the previous observations, we have moved on to examine how exactly a Poincaré map could be reconstructed. Additionally, based on our observation that the trajectory follows chaotic dynamics, it should be able to follow the requirements of a Poincaré map. Thus, a trajectory should be able to transverse the diagonal in a point *S*, which we will call the “entrance” point, and then it should return to the diagonal through another point *S′*, which we will call the “exit” point. A Poincaré map, in order to manifest non-chaotic orbits, requires that a dynamic system converges to its limit cycle (In a dynamic system of the form x′(t)=F(x(t)), with a function F such as: $F:{\mathbb{R}}^{2}\to {\mathbb{R}}^{2}$ and the trajectory of the function is also a smooth function x(t) with domain ${\mathbb{R}}^{2}$. Then, such a trajectory is called closed (or periodic) (meaning it is not constant but returns to its starting point) if there exists a *t*_0_ > 0 such as $x(t+t_{0})=x(t)\forall t\in \mathbb{R}$, ∀ t∈ℝ). This is an equilibrium point where *S* ≡ *S′*. On the contrary, in our experimental setup we did not observe such an evidence of a global non-chaotic behavior. The system converged in the first convolution of the trajectory (Figure 12A,B) as well as after cell culture media supplementation (Figure 12E). In the other cases the trajectory (Figure 12C,D,F–K) diverged from equilibrium indicating that a Poincaré cross-section followed chaotic dynamics.

**Figure 13:**
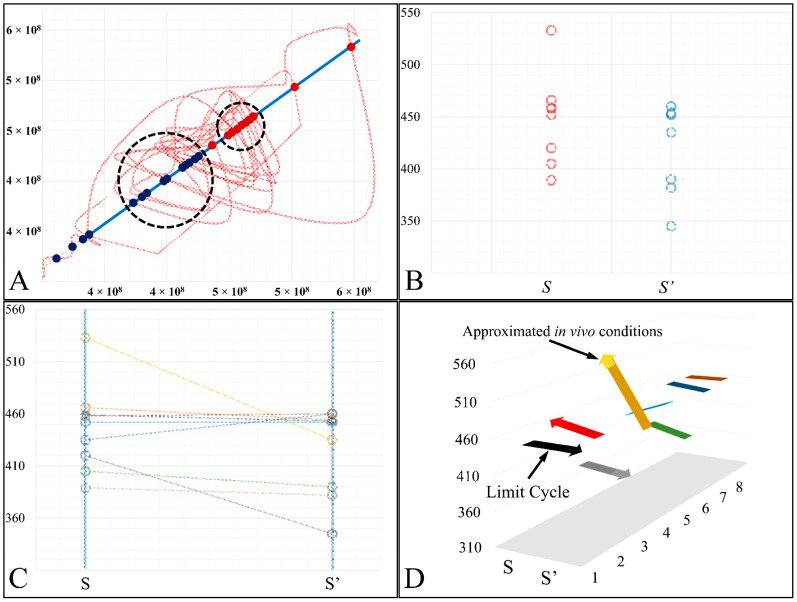
The *Poincaré* map cross-sections. We have presented all sections of the trajectory on the diagonal (**A**), along with the transmission points of *S*→*S*′ (**B**), as well as the respective points with their mapping from S onto S′ (**C**). Finally, the 3D representation of the cross-sections along with the cell populations is presented (**D**). The *x*-axis corresponds to the population of cells at time *t* and the *y*-axis corresponds to the population of cells at time *t* + 1.

In order to better understand the aforementioned dynamics, we have tried to illustrate the cross-sections of the Poincaré map, in a series of graphs. In particular, in Figure 13 we have presented all points of the trajectory that cross the diagonal (Figure 13A), which is also presented as a second phase-space of the *S→S′* points (Figure 13B,C). In particular, in the 3D representation the transition of the trajectory to its highest point became apparent, which corresponded to a point of cell culture media supplementation, resembling an in vivo situation of leukemic cells being provided with constant nutrients (Figure 13D).

## 4. Discussion

In the present work, we have attempted to provide a further insight into the dynamics of leukemic cells proliferation dynamics. Previous works, such that of Wolfrom et al., (2000), have studied the proliferation dynamics of adherent cells and in agreement to our study have also found that cells do follow chaotic orbits. In these previous works, the cell population at the end of a time period has been investigated and it was shown that cells grow in non-linear patterns [23,24,25]. All reports, to the best of our knowledge, concern the investigation of adherent cells, which correspond to solid tumors in an in vivo situation. Our study is the first that reports on the proliferation and chaos dynamics of leukemic cells.

Do cancer cells progress against the physiological norms? A normal eukaryotic cell is created, ages and dies. Cancer follows a different path, that of birth, transformation and immortality. If we examine the phenomenon from the systems theory point of view, it appears that cancer cells follow a distorted course and therefore “violates” the physiological pathways [43]. Yet, how is it to define “normal” and “abnormal” in terms of cellular physiology? How certain are we that carcinogenesis is just not another evolutionary cellular path and not an aberration. Assuming that there are some general rules concerning living organisms, the later follow some sort of pattern and therefore it can be assumed that cancer cells move outside of these patterns/motifs (or maybe not). Yet, a new question arises; are cancer cells that, if considered aberrant, live “illegally” within the patterns of life? One thing is certain, there are some fundamental laws of nature that apply to all living organisms and no kind of life is able to deviate from them [43]. Such examples include the law of conservation of energy and the laws of thermodynamics, which are derived from the laws of conservation of energy. At this point we could paraphrase the words of *Planck* and *Schrödinger* by adding, “… it is certain that the leopard will have a fur with polka dots, but what will be their pattern this is a probability…” [43]. In order to be able to predict a chaotic/non-linear phenomenon, we must know with infinite detail its initial conditions. However, this question can be reversed as, can we find the initial conditions knowing the final? Previous studies have reported that biological systems have complex dynamics [24,45]. To the best of our knowledge, no study has answered that question yet. This very complexity makes biological systems interesting and challenging.

In the present study we addressed the question of whether chaotic dynamics could be detected in the proliferation of acute lymphoblastic leukemia cells in in vitro cell culture. In vitro systems offer the ability to perform long-term experiments and isolate the system under study to reduce the complexity of in vivo systems [46,47]. Such studies are impossible to carry out in in vivo systems, both in terms of technical and experimental, as well as bioethical restrictions. Therefore, in vitro systems are ideal, and perhaps the only ones that allow, for the study of proliferation experiments, where the initial number of cells can be controlled. Another aspect that should be taken into account was the total experimental progress. In particular, we have referred to the “Materials and Methods” section in the process followed, which included the media replacement, centrifugation of cells and the re-substitution of cells in fresh media. This process was performed in order to simulate the in vivo conditions, where leukemic cells are under a constant supply of nutrients. On the other hand, centrifugation could probably affect cell proliferation, yet it was impossible to do otherwise since the metabolic waste and starvation stress could affect the experimental conditions drastically. Yet, removing cell media and replenishing the cells with fresh one, poses two possibilities. First, cells could be stressed in such a way that it would disrupt their progression and second, cells could have a sort of “memory” mechanism to assist them in continuing their previous proliferation. It is possible that the present cellular model is closer to the second hypothesis, as after each media change cells continued to proliferate almost with the same rate. For example, media were changed at day 21 and at day 22 cells moved from the G1 phase to S-phase, indicating cells continued proliferating as they probably manifested a sort of “memory” of their previous state. Further on, it is possible that cells do not die silently to other cells, but by releasing lysosomes that can harm surrounding cells. Thus it is possible that changing the media mostly obviate the expected inflammatory environment which occurs *in vivo*. In addition, it is possible that circulating leukemic cells derive from the bone marrow which approximates a solid milieu. his should be stated. As aforementioned, our cellular model it is known to manifest autocrine catalytic signalling, indicating that extracellular releasing of lysosomes is possible. In our cellular model we had to change the media, in order to renew nutrient, as well as remove toxic metabolic waste. This was the closest way we could think of for approaching in vivo conditions as much as possible. On the other hand, although the in vivo conditions can produce inflammatory conditions they have at the same time “salvation” mechanisms, which can alleviate the inflammatory effects.

Cancer is known to start and progress slowly, at least before the clinical diagnosis. Knowledge on the mechanisms of progression before the onset of clinical symptoms can contribute to the timely treatment of serious diseases, such as tumors. As aforementioned, the main question is whether we can predict the course of a tumor from the time of its appearance to the point of clinical presentation [29,30,43,44,48,49]. The only way to diagnose cancer, up to date, is by its symptoms. Preventive examination is available, but for a small number of malignancies. In addition, our knowledge is very restricted with respect to the time between the onset of the first cancer cells and the point of diagnosis [29,30,43,44,48,49].

Cell cultures are thought to follow a linear growth pattern. Since the sigmoid function-equation takes into account the limitation in resources i.e., food and space, it predicts that a population of cells will reach a steady state within a certain period of time and cells eventually die or cease to proliferate. In our model, we have introduced a new constant, making food readily available but keeping the space constant, relative to the total volume within which cells were allowed to grow. Under these conditions, we derived the time series of cell proliferation, which showed aperiodic oscillations. The transformation of the (aperiodic) time series to a phase space, the (positive) Lyapunov indices, as well as the Poincaré representations we performed, confirmed the chaotic behavior of this dynamic system. Our work provided evidence for deterministic chaos in the proliferative behavior of leukemia cells in vitro. Given that this is a very complex phenomenon, much more effort and studies are required in order to understand the underlying mechanisms. The implications from understanding such dynamic systems are immense. Such experimental approaches could provide us with insight on disease progression, such as cancer, and as a consequence would allow us to model the disease in real-life situations.

Although, we have suggested that in vitro systems, are ideal for the questions posed in the present study, they also pose some limitations. First of all, in vitro systems cannot simulate the in vivo environment and in particular, the tumor microenvironment, which is known to play a significant role in tumor progression. Simulating the tumor microenvironment is a very tedious task. There are numerous factors that play a role, including growth and inflammatory factors, which are difficult to study in an in vitro system. Another significant limitation is the measurement of very small cell populations. For example, it would be very interesting to simulate tumor ontogenesis, in vitro, starting from as few as one or two cells. Yet, due to methodological limitations it is not possible to measure the cell proliferation until cells reach a critical point, where methodological approaches are able to determine the cell population.

Thus, based on the aforementioned limitations, future efforts could focus on adding factors (for instance growth factors) to simulate the tumor microenvironment, and in particular, one factor at a time. Another interesting approach could focus on the investigations of methods that could measure very small cell populations and thus facilitating tumor progression at the very first stages of growth. Finally, these recommendations could be investigated under the presence of chemotherapeutics (for example glucocorticoids, which are first line agents in the treatment of childhood leukemia).

## 5. Conclusions

Tumor cell proliferation is considered one of the most important properties to consider for the diagnosis and treatment of cancer. One basic question, concerning cancer, still remains unsolved. How much time does it take for a tumor to be diagnosed beginning from the day the first tumor cells appear. In other words, what happens from the emergence of the first cancer cells to the clinical presentation? This question is difficult to answer. The only way to solve this problem is by understanding the proliferation dynamics of tumor cells and attempt to predict the phenomenon. Towards that end, in vitro models can prove very useful since they allow us to perform multiple experiments from desirable low cell populations and observe their proliferation patterns.

In that sense, we have made the observation that leukemic cells follow chaotic orbits, in vitro, as well as their population dynamics is dependent on initial conditions. Thus, we have presented evidence that cell proliferation progresses differently, dependent on the initial cell population. Based on this observation, it is possible that it would be feasible to estimate both the initial number of cells as well as the time needed for the leukemic cells to become diagnosed (in our case, produce a dense population resembling that of the clinical presentation).

Our understanding of tumor cell proliferation dynamics is still very limited. We believe that our approach could prove useful towards the understanding of cell proliferation mechanics and as such it consists for us an ongoing research topic.

## Data Availability

Data are available from the corresponding author upon reasonable request.

## Supplementary Materials

The following are available online at https://www.mdpi.com/article/10.3390/cells10123584/s1, Figure S1: We have separated the growth curves for both the initial populations i.e., 20 cells/μL (A) and 200 cells/μL (B) into four phases: the first phase included the exponential growth (designated as “exp” and colored with blue), the logistic growth (designated as “log” and colored with red), the logarithmic growth (designated as “logistic” and colored with purple) and a polynomial growth (designated as “poly” and colored with grey). These regressions consisted approximations of the total growth curve, therefore we have attempted to investigate the detailed non-linear dynamics for chaotic orbits. This analysis was performed by regressing each separate phase with known functions, whose *R*^2^ values are presented for each respective curve (using the same coloring scheme). Although regressions were performed, they still concerned an approximation of the growth curves, and therefore our intention was to investigate the details of the manifested non-linear dynamics with more specialized tools, Table S1: Cell Proliferation Data, Video S1: Animated Figure 11.
