# Supplementary material for: Poincaré Maps and Aperiodic Oscillations in Leukemic Cell Proliferation Reveal Chaotic Dynamics
Source: Cells. 2021 Dec 19;10(12):3584. doi: 10.3390/cells10123584 (PMC8700028; doi:10.3390/cells10123584)
Supplement: Supplementary file 1 [file cells-10-03584-s001.zip › Figure S1.pdf]

cells

Article

Poincare Maps and Aperiodic Oscillations in Leukemic Cell Proliferation Reveal Chaotic Dynamics

Konstantinos Adamopoulos [1], Dimitis Koutsouris [1], Apostolos Zaravinos [2,3,*] and George I. Lambrou [1,4,*]

1 Biomedical Engineering Laboratory, School of Electrical and Computer Engineering, National Technical University of Athens, Heroon Polytechneiou 9, 15780, Zografou, Athens, Greece; kadamopoulos@biomed.ntua.gr, dkoutsou@biomed.ntua.gr
2 Department of Life Sciences, School of Sciences, European University Cyprus, Diogenis Str. 6, Nicosia, 2404, Cyprus; a.zaravinos@euc.ac.cy
3 Cancer Genetics, Genomics and Systems Biology group, Basic and Translational Cancer Research Center (BTCRC), European University Cyprus, Nicosia 1516, Cyprus, email: a.zaravinos@euc.ac.cy
4 Choremeio Research Laboratory, First Department of Pediatrics, National and Kapodistrian University of Athens, Thivon & Levadeias 8, Goudi, 11527 Athens, Greece; glamprou@med.uoa.gr
* Correspondence: a.zaravinos@euc.ac.cy (A.Z.); glamprou@med.uoa.gr (G.I.L.); Tel. +974-4403-7819 (A.Z.); Tel. +302107467427 (G.I.L.)

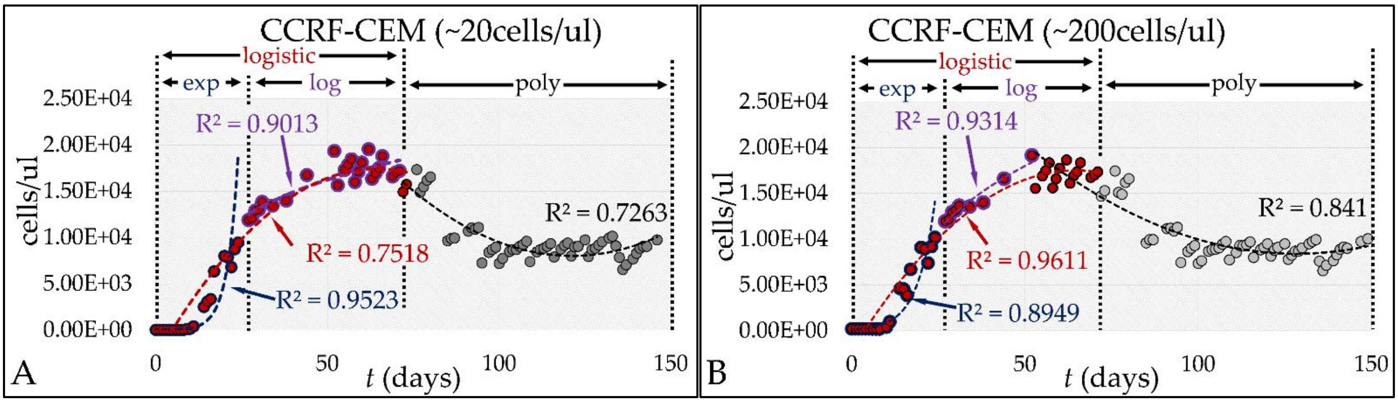

Figure S1. We have separated the growth curves for both the initial populations i.e. 20cells/ul (**A**) and 200cells/ul (**B**) into four phases: the first phase included the exponential growth (designated as "exp" and colored with blue), the logistic growth (designated as "log" and colored with red), the logarithmic growth (designated as "logistic" and colored with purple) and a polynomial growth (designated as "poly" and colored with grey). These regressions consisted approximations of the total growth curve, therefore we have attempted to investigate the detailed non-linear dynamics for chaotic orbits. This analysis was performed by regressing each separate phase with known functions, whose R^2 values are presented for each respective curve (using the same coloring scheme). Although regressions were performed, they still concerned an approximation of the growth curves, and therefore our intention was to investigate the details of the manifested non-linear dynamics with more specialized tools.
